# Supplementary material for: Controllable Carbonization of Plastic Waste into Three-Dimensional Porous Carbon Nanosheets by Combined Catalyst for High Performance Capacitor
Source: Nanomaterials (Basel). 2020 Jun 2;10(6):1097. doi: 10.3390/nano10061097 (PMC7353313; doi:10.3390/nano10061097)
Supplement: Supplementary file 1 [file nanomaterials-10-01097-s001.pdf]

nanomaterials

Supporting Information

Controllable Carbonization of Plastic Waste into Three-Dimensional Porous Carbon Nanosheets by Combined Catalyst for High Performance Capacitor

Xueying Mu [1,2], Yunhui Li [1,*], Xiaoguang Liu [3], Changde Ma [2], Hanqing Jiang [2], Jiayi Zhu [4], Xuecheng Chen [3,*], Tao Tang [2,*] and Ewa Mijowska[3]

1. School of Chemistry and Environmental Engineering, Changchun University of Science and Technology, Changchun 130022, China; xymu@ciac.ac.cn
2. State Key Laboratory of Polymer Physics and Chemistry, Changchun Institute of Applied Chemistry, Chinese Academy of Sciences, Changchun 130022, China; cdma@ciac.ac.cn (C.M.); hqjiang@ciac.ac.cn (H.J.)
3. Faculty of Chemical Technology and Engineering, West Pomeranian University of Technology, Piastow Ave. 42, 71-065 Szczecin, Poland; Xiaoguang.Liu@zut.edu.pl (X.L.); emijowska@zut.edu.pl (E.M.)
4. State Key Laboratory of Environment-friendly Energy Materials, School of Science, Southwest University of Science and Technology, Mianyang 621010, China; jyzhu@swust.edu.cn

* Correspondence: liyh@cust.edu.cn (Y.L.); xchen@zut.edu.pl (X.C.); ttang@ciac.ac.cn (T.T.); Tel: +86-431-8558-2361 (Y.L.); +48-091-449-6030 (X.C.); +86-431-8526-2004 (T.T.)

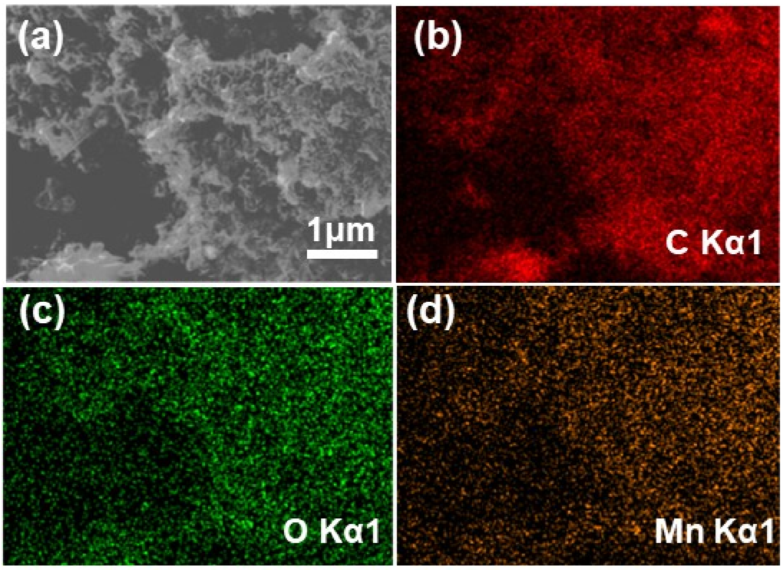

Figure S1. (a) SEM of PCS-MnO_2-2, EDX Mapping of (b) CKα1, (c) OKα1, (d) MnKα1.

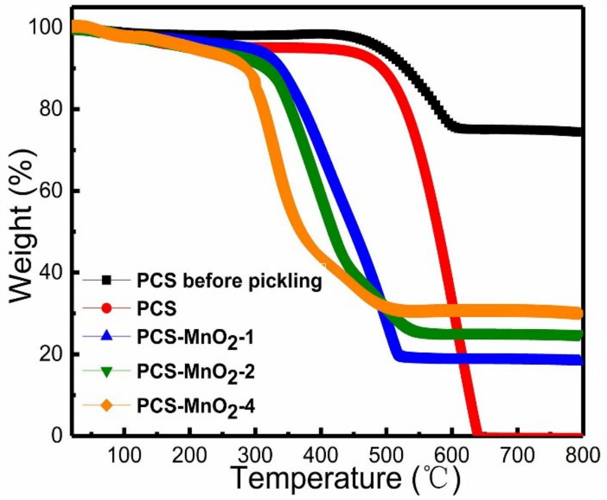

Figure S2. TGA curves of PCS-before pickling, PCS, and PCS-MnO_2 composites.

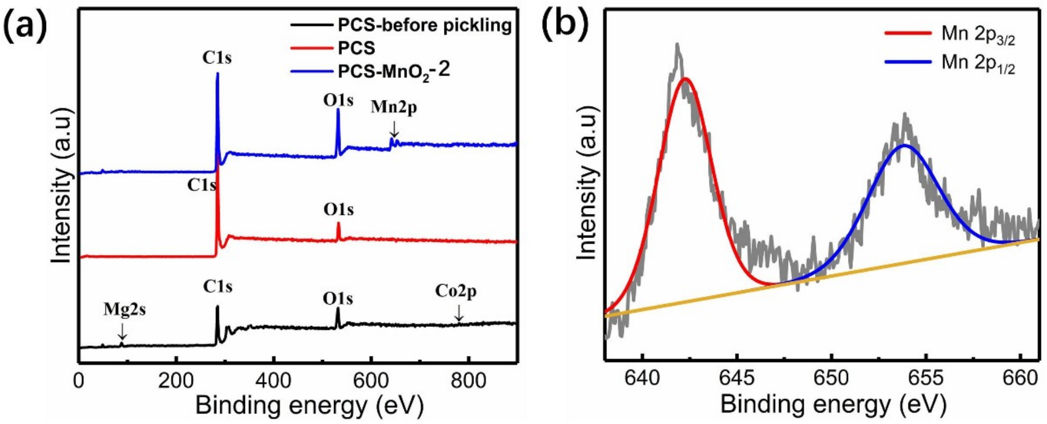

Figure S3. XPS survey spectra of (a). PCS-before pickling, PCS, PCS-MnO_2-2; (b)the narrow spectra of Mn_{2p} peaks of the PCS-MnO_2-2.

Table S1. of the capacitance retention of carbon materials in the literature.

Sample	Electrolyte	Test Condition	Capacitance	Capacitance Retentions	Reference
3D sponge N-doped graphene	6 M KOH	4 A g^{-1}	320 F g^{-1}	87.7%	[1]
Porous carbon nanosheets	6 M KOH	1 A g^{-1}	182 F g^{-1}	/	[2]
Porous activated carbons	2 M H_2SO_4	1 mA cm^{-2}	106~197 F g^{-1}	/	[3]
PCS-MnO_2	6 M KOH	10 A g^{-1}	64 F g^{-1}	90.1%	Current work

References

1. Elessawy, N.A.; El Nady, J.; Wazeer, W.; Kashyout, A.B. Development of High-Performance Supercapacitor based on a Novel Controllable Green Synthesis for 3D Nitrogen Doped Graphene. *Sci. Rep*. **2019**, *9*, 1129.
2. Wen, Y.; Kierzek, K.; Chen, X.; Gong, J.; Liu, J.; Niu, R.; Mijowska, E.; Tang, T. Mass production of hierarchically porous carbon nanosheets by carbonizing "real-world" mixed waste plastics toward excellent-performance supercapacitors. *Waste Manag*. **2019**, *87*, 691–700.
3. Domingo-Garcia, M.; Fernandez, J.A.; Almazan-Almazan, M.C.; Lopez-Garzon, F.J.; Stoeckli, F.; Centeno, T.A. Poly(ethylene terephthalate)-based carbons as electrode material in supercapacitors. *J. Power Sources* **2010**, *195*, 3810–3813.
